# Integrated Metabolome and Transcriptome Analysis Unveils Novel Pathway Involved in the Formation of Yellow Peel in Cucumber

**DOI:** 10.3390/ijms22031494

**Published:** 2021-02-02

**Authors:** Chen Chen, Geng Zhou, Juan Chen, Xiaohong Liu, Xiangyang Lu, Huiming Chen, Yun Tian

**Affiliations:** 1 College of Bioscience and Biotechnology, Hunan Agricultural University, Changsha 410128, China; yb20160012@stu.hunau.edu.cn (C.C.); xiangyangcn@163.com (X.L.); 2 Hunan Vegetable Research Institute, Hunan Academy of Agricultural Sciences, Changsha 410125, China; zhougeng@hnu.edu.cn (G.Z.); chenj84691746@163.com (J.C.); vseed@163.com (X.L.); 3 Longping Branch, Graduate School of Hunan University, Changsha 410125, China

**Keywords:** cucumber peel color, peel yellowing, metabolome, transcriptome

## Abstract

Yellow peel will adversely affect the appearance quality of cucumber fruit, but the metabolites and the molecular mechanism of pigment accumulation in cucumber peel remain unclear. Flavonoid metabolome and transcriptome analyses were carried out on the young peel and old peel of the color mutant L19 and the near-isogenic line L14. The results showed that there were 165 differential flavonoid metabolites in the old peel between L14 and L19. The total content of representative flavonoid metabolites in the old peel of L14 was 95 times that of L19, and 35 times that of young peel of L14, respectively. This might explain the difference of pigment accumulation in yellow peel. Furthermore, transcriptome analysis showed that there were 3396 and 1115 differentially expressed genes in the yellow color difference group (Young L14 vs. Old L14 and Old L14 vs. Old L19), respectively. These differentially expressed genes were significantly enriched in the MAPK signaling pathway–plant, plant–pathogen interaction, flavonoid biosynthesis and cutin, suberine and wax biosynthesis pathways. By analyzing the correlation between differential metabolites and differentially expressed genes, six candidate genes related to the synthesis of glycitein, kaempferol and homoeriodictyol are potentially important. In addition, four key transcription factors that belong to R2R3-MYB, bHLH51 and WRKY23 might be the major drivers of transcriptional changes in the peel between L14 and L19. Then, the expression patterns of these important genes were confirmed by qRT-PCR. These results suggested that the biosynthesis pathway of homoeriodictyol was a novel way to affect the yellowing of cucumber peel. Together, the results of this study provide a research basis for the biosynthesis and regulation of flavonoids in cucumber peel and form a significant step towards identifying the molecular mechanism of cucumber peel yellowing.

## 1. Introduction

Cucumber (*Cucumis sativus* L.), which belongs to the annual herb of muskmelon of Cucurbitaceae, is an economically important crop worldwide [1]. Cucumber is native to India, but now widely planted in temperate and tropical regions all over the world [2,3,4,5]. Peel is the boundary of fruit, which is of great significance for maintaining fruit integrity and protecting it from external environment. Because there are many secondary metabolites in the peel, such as pigments, tannins and aromatic compounds, the peel can show a variety of colors [6,7]. Peel color is an important standard of food cucumber quality traits, which can greatly affect consumers’ choices, so it has an important commodity value [8]. The commodity value of cucumber peel color has strong regional characteristics, so consumers in different regions have different preferences for it, and it is impossible to use a unified standard to judge the quality of a certain peel color. Therefore, combining with the market demands of cucumber, it is an important breeding goal to improve the economic value through perfecting peel color.

The peel color is a quantitative character, and its gene expression is affected by the environment [8]. Hutchins first classified the peel color of mature cucumber fruit into four categories: red, orange, yellow and cream, and proposed it was controlled by two genes *R* and *C* [9]. Subsequently, the related research studies are few. At present, only five genes related to peel color trait have been identified in cucumber. *CsaARC5* and *CsYcf54* confer light green peel [10,11], *w* controls white peel [12], *CsMYB36* is involved in the formation of yellow green peel [8] and *CsMYB60* is related to orange peel [13]; these genes determine cucumber peel colors by regulating chlorophyll and flavonoid metabolic pathways. However, the research progress on the signal pathway involved in the formation of yellow peel in cucumber remains elusive.

In recent years, joint analysis based on multifunctional “omics” data has been demonstrated to be a powerful tool to clarify different aspects of plant developmental biology as well as environmental responses [14,15,16], such as drought stress response [4], effects of grafting different rootstocks on fruit flavor [1] and astringency-related gene mining in cucumber [17]. Furthermore, transcriptome and metabolome analysis have been widely used to identify signal pathway and mechanisms controlling pigment accumulation in plant pulp or peel. For example, an integrated analysis of the transcriptome and metabolome in sprouts germinated from three colored potato cultivars was performed; the results suggested that the connection network of anthocyanins and genes showed a regulatory system involved in the pigmentation of light-red Hongyoung and dark-purple Jayoung potatoes [14]. Combined metabolomic and transcriptomic analyses were carried out with fig cultivar green peel and its color mutant purple peel, and five and twenty-two metabolites were identified as having significantly different contents between fruit peels of the two cultivars at young and mature stages, respectively [7]. Similarly, the mechanisms of the differential anthocyanin accumulation in purple and green turnips were clarified by combined transcriptome and metabolome analysis, providing an important theoretical basis for further in-depth analysis of the candidate structural genes along with the key transcription factors predicted to modulate anthocyanins in turnip towards developing new turnip varieties with improved nutritional quality [18]. Network analysis of the metabolome and transcriptome has been applied as a powerful tool to investigate the novel genes and pathways in plants, so it is also effective for exploring the novel pathway involved in the formation of yellow peel in cucumber [19,20].

During the processes of cucumber variety breeding and planting, we noticed that the peel color of some cucumber varieties would become light yellow or yellow, especially in the ecotype cucumber varieties of South China type. This yellow appearance may be interpreted by consumers as aging or poor-quality fruit, making it unmarketable. In this study, we selected peel color mutant L19 and its near-isogenic line L14, based on the peel color, the contents of carotenoids and flavonoids. Then, we applied integrated metabolome and transcriptome tools, including network analysis based on correlations between metabolite and transcript levels. Our findings provide new insights into the molecular mechanisms of flavonoids accumulation in cucumber peel and highlight the usefulness of the integrated approach for clarifying this process.

## 2. Results

### 2.1. Phenotype of Two Near-Isogenic Cucumber Varieties

**Figure 1:**
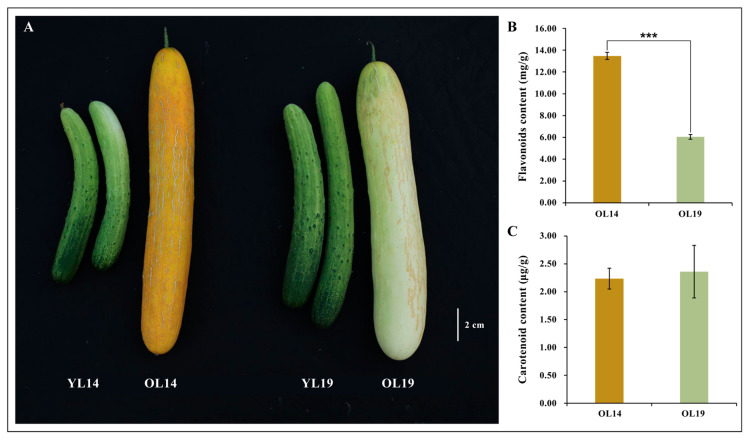
The phenotypic characters and determination of flavonoids and carotenoids in cucumber materials. (**A**) Phenotype of fruit in YL14, OL14, YL19 and OL19. Scale bar = 2 cm. Flavonoids content (**B**) and carotenoid content (**C**) in cucumber fruit at maturity. Bars are means of three replicates ± SEM. *** *p* < 0.001.

L14 and L19 are two near-isogenic cucumber varieties. In the early stage of ripening cucumber, the young peels of L14 and L19 were green-white color. In the summer with long day and high temperature, the peel of L14 is green-white with light yellow inlay, and the peel of L19 is green-white under the same environment. At the late stage of cucumber maturity, the peel color of L14 became yellow, while that of L19 was still white. We sampled peels of young and old cucumber fruits for both lines. Four types of samples were obtained: YL14 (L14 young peel is green-white), OL14 (L14 old peel is yellow), YL19 (L19 young peel is green-white), and OL19 (L19 old peel is white) (Figure 1A). Among them, YL14 vs. OL14 and OL14 vs. OL19 were two groups of peel samples with an obvious yellow color difference, while there was no significant yellow color difference between YL14 vs. YL19 and YL19 vs. OL19. Previous works show that plants can turn to yellow, orange-red or red due to accumulation of carotenoids and flavonoids [21,22]. Therefore, we respectively detected the average content of carotenoids and flavonoids in L14 and L19 dried peels of cucumbers at maturity. The results show that the average content of flavonoids is much higher than the carotenoids. No significant difference is detected on the averaged carotenoid content between L14 and L19 dried peels (2.24 μg/g vs. 2.36 μg/g, dry weight) (Figure 1C), while the total content of flavonoids in L14 was about 2.22 times higher than that of L19 (13.47 mg/g vs. 6.05 mg/g) (Figure 1B). So, the findings testified that the peel yellowing of OL14 could be due to the accumulation of flavonoids.

### 2.2. Differential Accumulation of Flavonoid Metabolites between L14 and L19

**Figure 2:**
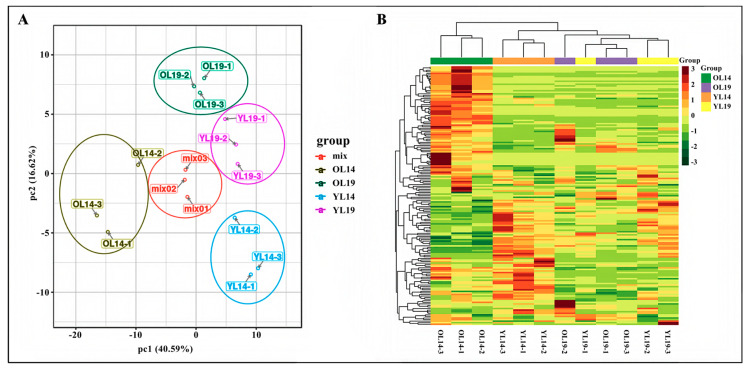
Differential flavonoid metabolite analysis on the basis of principal component (PCA) and clustering heat map. (**A**) PCA score map. (**B**) Clustering heat map of metabolites.

In total, 165 flavonoid metabolites were detected in YL14, OL14, YL19 and OL19 samples using an UPLC–MS/MS detection platform. These 165 flavonoid metabolites of 8 catechin derivatives, 13 anthocyanins, 48 flavones, 29 flavonols, 6 flavonolignans, 37 flavone C-glycosides, 15 flavanones, 8 isoflavones and 1 alkaloid (Supplementary Table S1). To better understand the overall metabolic differences in flavonoids among the samples and the degree of variation between the samples within the group [23], the samples were analyzed by principal component analysis (PCA). The results showed that principal component 1 (pc1) and principal component 2 are accounted for 40.59% and 16.62% of the total variation. Furthermore, OL14 (yellow) was clearly distinguished from the other three groups of samples in the pc1 and pc2 score plots, and the repeated samples were compactly gathered together, thus indicating that the experiment was reproducible and reliable (Figure 2A). R software (www.r-project.org/) was used to normalize the metabolite content data using the range method, and the difference in accumulated metabolites among different samples were analyzed and displayed using hierarchical cluster analysis (HCA). It was found that the changes of flavonoid metabolites in the OL14 group were significantly different from those in the other three groups of YL14, YL19 and OL19 (Figure 2B). These results suggest that there may be a large number of metabolites in OL14 related to the yellowing of cucumber peels.

**Figure 3:**
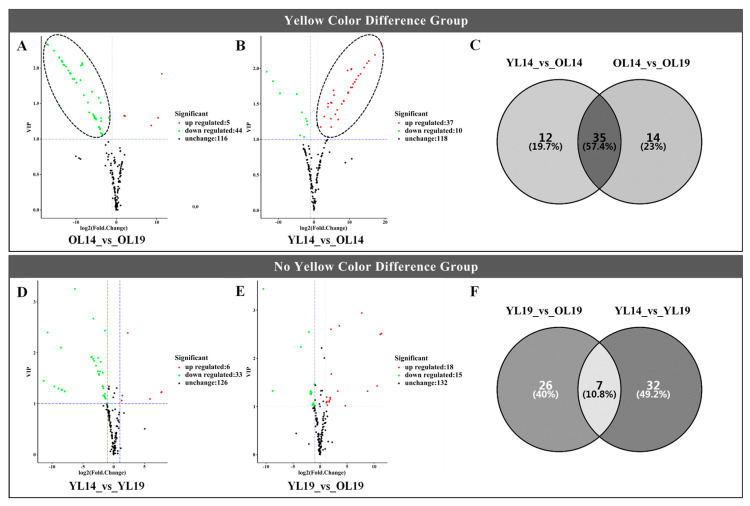
The metabolite volcano diagram and Wayne diagram. (**A**,**B**,**D**,**E**) The volcano map showed the difference in the expression level of metabolites in the four groups of samples, and the difference was statistically significant. The (**C**,**F**) Venn diagram depicts the common and unique number of (shared and unique) DAF among the four groups of samples, respectively.

Based on OPLS-DA results, the differentially accumulated flavonoids (DAFs) with significant differences were counted for the four groups (YL14_vs_OL14, OL14_vs_OL19, YL19_vs_OL19, YL14_vs_YL19) in the range of VIP (The Variable Importance in the Projection) ≥ 1, with Log2FC (multiple of difference for log2 treatment) ≥ 2 and Log2FC ≤ 0.5. In YL14_vs_OL14, there were 10 DAFs downregulated (It meant that there were 10 kinds of DAFs that were lower in OL14, compared to YL14) and 37 DAFs upregulated; YL19_vs_OL19 had 15 DAFs downregulated and 18 DAFs upregulated; OL14_vs_OL19 had 44 DAFs downregulated and 5 DAFs upregulated; YL14_vs_YL19 had 33 DAFs downregulated and 6 DAFs upregulated. Next, we checked composition of DAFs with significant differences within each of the comparing groups and the results were visualized using volcano plot. This reveals that the distribution of downregulated metabolites in OL14_vs_OL19 of yellow color difference group was symmetrical with that of upregulated metabolites in YL14_vs_OL14 (Figure 3A,B). While such pattern has not been observed in the no yellow color difference group (Figure 3D,E). Further analysis showed that there were 35 kinds of the same DAFs in the yellow color difference group YL14_vs_OL14 and OL14_vs_OL19, accounting for 57.4% (Figure 3C), while there were only 7 kinds of the same DAFs in the no yellow color difference groupYL14_vs_YL19 and YL19_vs_OL19, accounting for 10.8% (Figure 3F). It was found that 30 kinds of DAFs in the yellow color difference group were upregulated in OL14 and downregulated in OL19 and YL14 (Table 1). When the contents of each of the 30 DAFs were added together, the total content of flavonoid metabolites in OL14 was 95 times that of OL19 and 35 times that of YL14. These DAFs are divided into seven categories, including flavones, isoflavones, flavonols, flavanones, flavone C-glycosides, anthocyanins, catechin derivatives. In this study, the flavanones with high content in OL14 were naringenin O-malonylhexoside and naringenin. The isoflavones with high content in OL14 were 6-Hydroxydaidzein, glycitein and calycosin. Four substances in flavone C-glycosides (apigenin C-glucoside, isovitexin, naringenin C-hexoside, C-hexosyl-apigenin O-caffeoylhexoside) were detected in young cucumber YL14 and old cucumber OL14, but these four substances accumulated significantly in old cucumber OL14. Two flavonols (kaempferide and dihydrokaempferol) and two flavones (acacetin O-acetyl hexoside and sakuranetin) were accumulated only in OL14. The callistephin chloride in anthocyanins and the epicatechin gallate in catechin derivatives were detected in OL14. For most of these flavonoid metabolites, they showed existence/nonexistence patterns in OL14, OL19 and YL14, indicating that they are the key components leading to cucumber peels to turn yellow.

### 2.3. L14 and L19 Transcriptome Analysis

**Figure 4:**
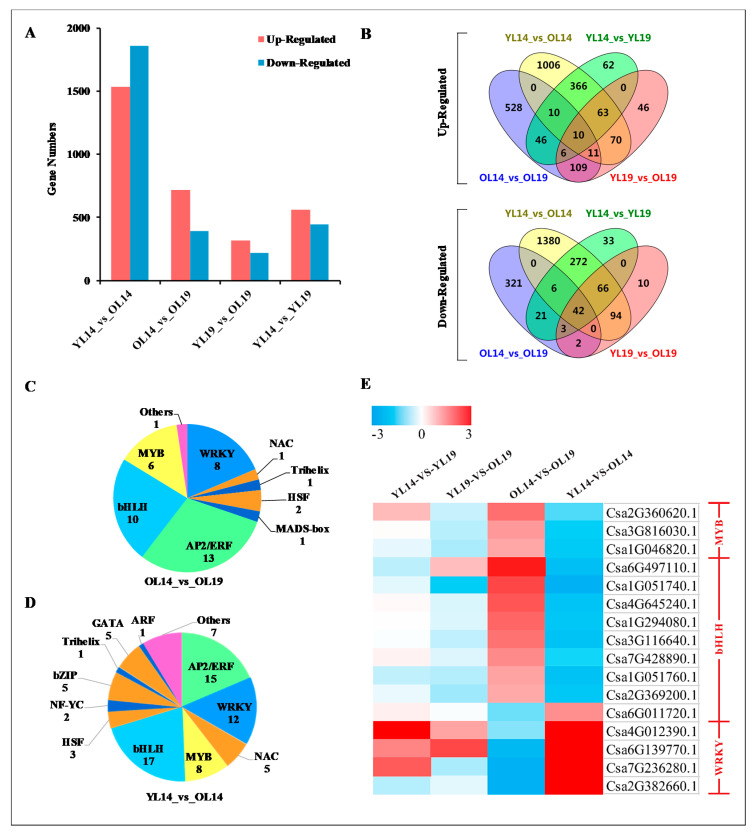
The statistics of the number of differentially expressed genes and cluster analysis of transcription factors. (**A**) Statistics of the number of upregulated and downregulated DEG in the four groups of samples. The Abscissa represents the comparison between samples (groups), and the ordinate indicates the significant differentially expressed genes detected. (**B**) The Venn diagram depicts the common and unique number of DEG among the four groups of samples. The quantitative distribution of differentially expressed TFs in OL14_vs_OL19 (**C**) and YL14_vs_OL14 (**D**). (**E**) The heat map shows the expression multiple changes (Log2 multiple changes) of the gene encoding transcription factor among four groups of samples.

We collected samples of YL14, OL14, YL19 and OL19 for RNA-Seq analysis. The quantitative results of all samples were obtained by filtering low expression genes, and significantly differentially expressed genes were identified according to the threshold of |log2 Fold Change| ≥ 1 and probability >0.8. In the comparison of YL14_vs_OL14, YL19_vs_OL19, YL14_vs_YL19 and OL14_vs_OL19, 3396 (upregulated 1536, downregulated 1860), 532 (upregulated 315, downregulated 217), 1006 (upregulated 563, downregulated 443), 1115 (upregulated 720, downregulated 395) significant differential expression genes (DEGs) were identified (Figure 4A,B; Supplementary Table S2). This shows that the molecular level between the samples with yellow color difference (YL14_vs_OL14 and OL14_vs_OL19) and those without yellow color difference (YL14_vs_YL19 and YL19_vs_OL19) was significant differences.

The Gene Ontology (GO) enrichment analysis of the above-mentioned DEGs was carried out, and the significantly enriched GO items in the differentially expressed genes were screened (Supplementary Figure S1). We compared the GO enrichment of DEGs in four groups: YL14_vs_OL14, YL19_vs_OL19, YL14_vs_YL19 and OL14_vs_OL19. It was found that DEGs for all four comparisons were significantly enriched in metabolic process, cellular process and single-organism process in biological processes. Membrane, membrane part, cell and cell part are the main enrichment items of DEGs in cellular components. In the category of molecular functions, DEGs are mainly enriched in binding and catalytic activity. To further understand the biological function of DEGs, we applied pathway significant enrichment analysis to identify metabolic pathways and signal pathways (Supplementary Table S3). The results showed that DEGs in YL14_vs_OL14, YL14_vs_YL19 and OL14_vs_OL19 were enriched in the metabolic pathways, biosynthesis of secondary metabolites pathway, phenylpropanoid biosynthesis pathway, photosynthesis pathway and photosynthesis-antenna proteins pathway. Only OL14_vs_OL19 had DEGs that enriched in the phenylalanine metabolism pathway. These DEGs in the yellow color difference group (YL14_vs_OL14 and OL14_vs_OL19) were significantly enriched in the MAPK signaling pathway-plant, plant-pathogen interaction, flavonoid biosynthesis and cutin, suberine and wax biosynthesis pathways. In conclusion, DEGs analysis shows that the cucumber peel yellowing in OL14 might be due to differentially expressed genes involved in these biological metabolic processes.

Transcription factors (TFs), also known as trans-acting factor, regulates plant growth and development, environmental stress response and biosynthesis of secondary metabolites by activating or inhibiting gene expression [24,25]. A total of 150 differentially expressed TFs were identified in L14 and L19 (Supplementary Table S4). These TFs were distributed in 8 gene families, including AP2/ERF (38), WRKY (32), bHLH (29), MYB (14), NAC (6), HSF (5), bZIP (5) and GATA (5) (Supplementary Table S5). Most of the TFs were concentrated in the peel yellow color difference groups (OL14_vs_OL19 and YL14_vs_OL14), which contained 43 and 81 differentially expressed TFs, respectively (Figure 4C,D). Transcription factors WRKY, MYB and bHLH play an important role in the structural and genetic regulation of plant pigment (flavonoid-anthocyanin) biosynthesis [18]. Therefore, we further analyzed the TFs of the three families and found that WRKY (*Csa6G139770.1*) and bHLH (*Csa6G011720.1*) were significantly upregulated in OL14, while MYB (*Csa3G816030.1*, *Csa1G046820.1*), which was significantly downregulated in OL19, showed the opposite trend (Figure 4E), indicating that these four transcriptional factor coding genes might be the main regulatory genes for cucumber peel yellowing.

### 2.4. Association Analysis of Genes and Metabolites Related to Yellowing of Cucumber Peel

**Figure 5:**
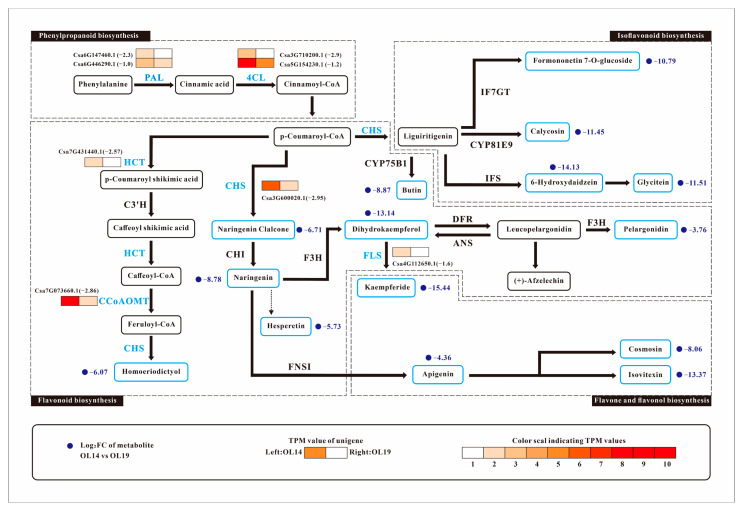
OL14_vs_OL19 changes in the expression of phenylpropionic acid, flavonoids, isoflavones, flavonoid and flavonol biosynthesis pathway genes and metabolites. OL14 and OL19 are old cucumber fruits. OL14, peel is yellow; OL19, peel is white. TPM: transcripts per million.

The old peel of cucumber OL14 and OL19 show obvious yellow color difference. DAFs and DEGs in OL14_vs_OL19 were mapped to the corresponding KEGG (http://www.genome.jp/kegg/) pathway to reveal the relationship between key genes and metabolites associated with cucumber peel yellowing (Table 1 and Table 2), and the network map of genes and metabolites was drawn (Figure 5). We identified 8 DEGs involved in the biosynthesis of phenylpropanoid, flavonoid, isoflavonoid, flavone and flavonol. We found that the downregulation of these genes in OL19 led to decrease in the content of a large number of flavonoid metabolites. Compared with OL14, the two phenylalanine ammonia-lyase genes (*PAL*) (*Csa6G147460.1*, *Csa6G446290.1*) at the entrance of the phenylpropanoid biosynthesis pathway were downregulated by 2.3 times and 1.0 times in OL19, respectively. PAL is the key enzyme that catalyzes the conversion of phenylalanine to cinnamic acid [26,27,28]. It was found that UV radiation could induce the increase of PAL activity and promote the synthesis of related secondary metabolites [29]. Two 4-coumarate CoA ligase genes (*4CL*) (*Csa3G710200.1*, *Csa5G154230.1*) were downregulated by 2.9 times and 1.2 times in OL19, respectively. 4CL catalyzes cinnamic acid to produce corresponding coenzyme A lipids, and these intermediate products are then involved in different phenylpropanoid biosynthesis pathways [30,31]. Chalcone synthase gene (*CHS*) (*Csa3G600020.1*, 2.95 times downregulated) is the key gene in the phenylpropanoid biosynthesis pathway to flavonoid biosynthesis pathway. CHS provides the basic carbon frame structure for flavonoids and is a committed step for the synthesis of flavonols, flavanones and other substances [32]. The biological substrate of CHS is derived from coumaroyl-CoA in the phenylpropanoid biosynthesis pathway. Under stress conditions, the biological substrate of CHS can be cinnamoyl-CoA or caffeoyl-CoA [33]. *HCT* (*Csa7G431440.1*) is downregulated by 2.57 times in OL19. It encodes shikimate O-hydroxycinnamoyltransferase to catalyze coumaroyl-CoA to caffeoyl-CoA [34]. The caffeoyl-CoA O-methyltransferase gene (*CCoAOMT*) (*Csa7G073660.1*) was downregulated by 2.86 times in OL19. CCoAOMT uses caffeoyl-CoA as substrate to form feruloyl-CoA [35,36]. Regulating the expression of *CCoAOMT* will affect the content of lignin and thus affect the stress resistance of plants [37,38]. Flavonol synthase encoded by *FLS* (*Csa4G112650.1*, 1.6 times downregulated) can catalyze dihydroflavonol to produce corresponding flavonol (Kaempferide). In order to better understand the relationship between genes and metabolites, genes and metabolites with the Pearson correlation coefficient greater than 0.8 in flavonoid, isoflavonoid, flavone and flavonol biosynthesis pathway were selected for correlation analysis. The results showed that there was a strong correlation between *CCoAOMT* (*Csa7G073660.1*) and Naringenin O-malonylhexoside in flavanone, kaempferide and dihydrokaempferol in flavonol, and 6-Hydroxydaidzein in isoflavone.

### 2.5. Validation of Transcriptomic Data Accuracy by qRT-PCR

**Figure 6:**
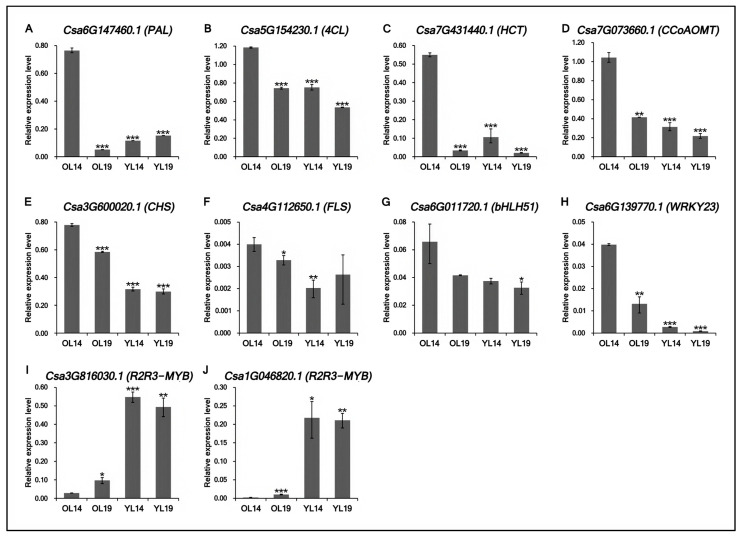
qRT-PCR analysis of the expression of differentially functional genes and transcription factors. (**A**–**F**) The relative expression levels of six functional genes (*Csa6G147460.1*, *Csa5G154230.1*, *Csa7G431440.1*, *Csa7G073660.1*, *Csa3G600020.1* and *Csa4G112650.1*). (**G**–**J**) The relative expression levels of four transcription factor coding genes (*Csa6G011720.1*, *Csa6G139770.1*, *Csa3G816030.1* and *Csa1G046820.1*). OL14, L14 old peel; OL19, L19 old peel; YL14, L14 young peel; YL19, L19 young peel. The internal normalization gene was *CsActin*. Bars are means of three replicates ± SEM. Asterisks (*) indicate the statistical significance of the difference between the experimental groups (OL19, YL14 and YL19) and the control group (OL14). (* *p* < 0.05, ** *p* < 0.01, *** *p* < 0.001).

Six DEGs and four TFs with higher correlation with peel yellowing were selected and verified by the qRT-PCR method. The results showed that the expression levels of six functional genes (*Csa6G147460.1*, *Csa5G154230.1*, *Csa7G431440.1*, *Csa7G073660.1*, *Csa3G600020.1* and *Csa4G112650.1*) (Figure 6A–F) and two transcription factor coding genes (*Csa6G011720.1* and *Csa6G139770.1*) (Figure 6G,H) in yellow old peel OL14 were higher than those in the other three samples. Compared with OL19, gene *Csa6G147460.1* (*PAL*) was upregulated nearly 15 times in OL14, and *Csa7G431440.1* (*HCT*) was upregulated nearly 16 times. The expression of two R2R3-MYB transcription factors (*Csa3G816030.1* and *Csa1G046820.1*) (Figure 6I,J) in OL14 was much lower than that in the other three samples. These results are consistent with the RNA-Seq data, indicating that the data can be used to evaluate the upregulation and downregulation of gene expression.

## 3. Discussion

The color of peel is not only an important quality standard, but also a key parameter affecting the marketability and consumer acceptance of fresh fruit products [39]. High temperature during the harvest of cucumbers often causes yellowing of cucumber peel, which strongly affects the fruit appearance quality of South China Type with light color peel. In this study, the candidate genes affecting cucumber peel yellowing were effectively explored by the combination of metabolic group and transcriptome. Previous studies show that the contents of chlorophyll, carotenoids and flavonoids are the main metabolic contents affecting color of plants. For example, in pepper (*Capsicum annuum* L.) [40], tomato (*Lycopersicon esculentum* Mill.) [41] and Arabidopsis (*Arabidopsis thaliana*) [42], transcription factor *GOLDEN2-LIKE* (*GLK*) regulating chlorophyll level. *CYC-B*, which was identified in orange tomatoes, is also functioning in regulating carotenoid content [43]. *SiMYB12* in pink tomato plays an important role in regulating flavonoids [44,45]. In this study, we found that the content of carotenoids in the dried peel of cucumber fruit maturity in L14 and L19 was low and had no significant difference, while the content of flavonoids increased significantly in the yellow peel of L14 (Figure 1). It was preliminarily concluded that flavonoids were the main reason for the yellowing of cucumber peel. Using the two near-isogenic lines of cucumber, the molecular basis of cucumber peel yellowing was studied.

Flavonoids are the largest type of secondary metabolites, which are widely distributed in many plants [46,47]. Flavonoids are not only the main compounds that determine the color of flowers, fruits and leaves, but also play an important role in plant growth, development and environmental adaptation [48,49,50,51]. Flavonols, flavanones and isoflavones are all important subclasses of flavonoids, which are widely found in common vegetables and fruits [52]. Previous studies have shown that the main pigment components in cucumber black thorn and orange peel are flavonol and proanthocyanidins [53]. The yellow peels of fruits such as lemons, oranges [54], limes, grapes and tomatoes are often rich in flavanones [52,55]. Based on UPLC–MS/MS detection platform, self-built database and multivariate statistical analysis, we analyzed the difference in flavonoid metabolites between young peel and old peel of L14 and its near-isogenic line mutant L19. Data analysis showed that there were 30 types of flavonoids with significant differences between young peel and old peel of cucumber varieties L14 and L19 (Table 1). The total content of these flavonoid metabolites in L14 old peel was 95 times higher than that of L19 old peel and 35 times higher than that of L14 young peel. They were significantly downregulated in OL14_vs_OL19 but upregulated in YL14_vs_OL14, which may explain the difference in peel color between L14 and L19. 

At present, more and more genes related to flavonoid biosynthesis pathway have been identified in some plant species: *PsDFR*, *PsANS* and *PhCHS* in peony [15,56]; *CitCHS* in citrus [47,57]; *MiCHS*, *MiCHI* and *MiF3H* in mango [58]; *SbCHS*, *SbCHI* and *SbF3H* in *Selaginella officinalis* [59]; *CHS-A*, *CHS-B* and *AcFLSs* in onion [60]; *AgMYB1* in celery [61]; *GbFLS* in ginkgo biloba [62,63]; *SmCHS*, *SmCHI* and *SmFNS* in *Salvia miltiorrhiza* [50]; *MaCHS* in mulberry [64]; *CtC4H2*, *CtCHS3*, *CtCHI3*, *CtF3H3* and *CtF3H1* in safflower [65]; *CsMYB6A* and *CsUGT72AM1* in purple leaf tea [66]; *AaFLS1* in *Artemisia annua* [67]. In order to explore the candidate genes that affecting cucumber peel yellowing, we further analyzed six DEGs involved in phenylpropionic acid, flavonoid, isoflavonoid, flavone and flavonol biosynthesis according to the relevant network map (Figure 5). *PAL* and *4CL* are two key enzyme genes in the phenylpropionic acid pathway in plants [68]. Coumaroyl-CoA, a branching point metabolite, can be produced under the catalysis of PAL and 4CL, which can be converted into naringenin chalcone or caffeoyl-CoA, which are substrates for the synthesis of flavonoids and tannins, respectively [69]. Xu et al. found that the expression of a PAL gene (*Csa1G590300.1*) in peel was significantly higher than that in the pulp, and three 4CL genes (*Csa1G050280.1*, *Csa1G050290.1* and *Csa1G108800.1*) may be involved in cucumber catechin biosynthesis [17]. Our data showed that *PAL* (*Csa6G147460.1*) and *4CL* (*Csa5G154230.1*) were enriched in the yellow peel of OL14 and downregulated in the white peel of OL19. It is showed that *PAL* and *4CL* are important genes in the upstream of the yellow regulatory network in cucumber peel.

**Figure 7:**
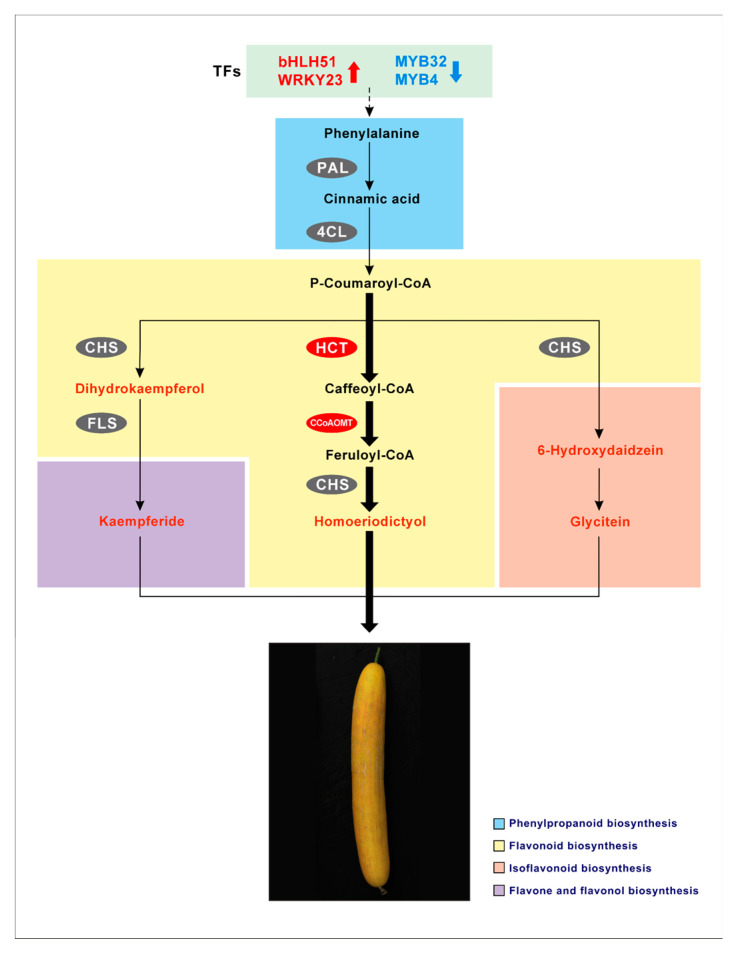
Prediction new model of cucumber peel yellowing. The red arrow indicated up-regulated. The blue arrow indicated down-regulated. TFs, Transcription Factors. PAL, Phenylalanine ammonia-lyase; 4CL, 4-coumarate-CoA ligase; HCT, Shikimate O-hydroxycinnamoyltransferase; CCoAOMT, Caffeoyl-CoA O-methyltransferase; CHS, Chalcone synthase; FLS, Flavonol synthase.

Chalcone synthase (CHS) was the first enzyme to be identified in the biosynthesis of flavonoids [70]. *CHS* is an important regulation gene located at the upstream point of the flavonoid biosynthesis pathway [71]. Overexpression of *CHS* may have a positive effect on the expression of *CHI* gene downstream of *CHS*, but it has no significant effect on *PAL* gene upstream of *CHS* in the flavonoid biosynthesis pathway [47]. In our predicted cucumber peel yellowing model, the enzyme encoded by *CHS* (*Csa3G600020.1*) plays an active role in the synthesis of at least three terminal flavonoid metabolites (Figure 7). Firstly, CHS controls the first step of flavonoid biosynthesis, catalyzing the synthesis of naringenin chalcone from p-Coumaroyl-CoA, and then rapidly converting to naringenin (flavanone) through chalcone isomerase (CHI) [72,73]. Then dihydrokaempferol was synthesized using naringin as substrate and kaempferide was synthesized under the action of enzyme encoded by *FLS* (*Csa4G112650.1*). Secondly, CHS can also catalyze p-Coumaroyl-CoA to produce liguiritigenin, and further synthesize 6-hydroxydaidzein through downstream enzymes involved in the isoflavone biosynthesis pathway, and finally form glycitein. Thirdly, CHS, as a downstream enzyme of the flavonoid biosynthesis pathway, catalyzes feruloyl-CoA to produce homoeriodictyol. In addition, combined with the results of qRT-PCR verification, we also found that the expression of *HCT* (*Csa7G431440.1*) and *CCoAOMT* (*Csa7G073660.1*) in the biosynthesis pathway of flavonoids, which is the branch of flavonoid biosynthesis pathway, in OL14 was significantly higher than that in OL19. Some studies have shown that *PhCCoAOMT1* plays an important role in benzene-propylene biosynthesis pathway in *Petunia hybrida*, and the downregulation of this gene can promote anthocyanin accumulation [74]. Our data show that there is a strong correlation between the expression of *CCoAOMT* and the accumulation of most terminal flavonoid metabolites, so we predict that the biosynthesis pathway of homoeriodictyol where *CCoAOMT* is located is an important pathway affecting cucumber peel yellowing, but whether the expression of this gene can promote the accumulation of flavonoids except anthocyanins remains to be confirmed by further experiments.

MYB and bHLH (basic helix-loop-helix) TFs exist in all eukaryotes, and they are the largest two families of plant transcription factors [75]. AtWRKY23 in *Arabidopsis thaliana* (L.) can stimulate flavonoid biosynthesis by upregulating enzyme encoding genes involving in flavonoid biosynthesis pathway [76]. The abnormal expression of bHLH3 in mulberry fruits will destroy the balance of flavonoid metabolic network, resulting in changes in the content and proportion of anthocyanins, flavonoids and flavonols in different colors of mulberry fruits [77]. It has been shown that transcription factors such as MYB, bHLH protein and WD repeat protein can not only form ternary complex MBW to regulate flavonoid accumulation in plants, but also be regulated independently of the ternary complex [46,78]. In the study of turnip pigment, MYB and bHLH positively regulated anthocyanin accumulation, while WRKY negatively regulated anthocyanin accumulation [18]. In our study, we found that both bHLH51 (*Csa6G011720.1*) and WRKY23 (*Csa6G139770.1*) accumulated in the yellow peel of OL14 and downregulated in the white peel of OL19. WRKY23 (*Csa6G139770.1*) is homologous to AtWRKY23. These two TFs may be involved in the positive regulation of cucumber peel yellowing. In addition, R2R3-MYB transcription factors are the largest class of MYB, which are divided into 25 subgroups [79]. R2R3-MYB has either positive or negative regulatory effect on the biosynthesis of plant flavonoids [61]. Five R2R3-MYB were upregulated in OL14_vs_OL19, of which two R2R3-MYB (*Csa3G816030.1*, *Csa1G046820.1*) were homologous to MYB4 and MYB32 in the fourth subfamily. Previous studies have shown that MYB4, MYB32, MYB7 and MYB3 in the fourth subgroup inhibit flavonoids by interacting with bHLH proteins [80,81]. Overexpression of *AtMYB4* can inhibit the expression of genes *CHS* and *4CL3* and hinder the synthesis of flavonoids induced by UV-B [82]. It is speculated that the accumulation of more two R2R3-MYB in OL19 may be involved in the negative regulation of cucumber peel yellowing.

## 4. Materials and Methods

### 4.1. Plant Materials

During the propagation of high generation inbred line parent Han203 (peel yellowing) in 2011, a mutant L with no yellowing peel was found. After years of systematic breeding, 5 generations of self-homozygous mutant L19 (green-white peel) and 5 generations of self-homozygous wild type L14 (yellow peel) were obtained. The F_1_, which is L19 crossed with L14, was obtained in the autumn of 2016 in Hunan, and the F_2_ generation was obtained by F_1_ self-cross in the winter of Hainan. In 2017, the parents’ lines, F_1_ and F_2_ generations were planted in the spring greenhouse in Hunan. During the fruiting period, the greenhouse was sealed to increase the temperature, which was beneficial to the formation of yellow cucumber peel. According to the comprehensive identification of cucumber color changes of young cucumber and old cucumber, the characters were investigated and genetic analysis was carried out. All the plants are planted in the greenhouse of Hunan vegetable Research Institute in Changsha. The cucumber fruits of L14 and L19 were harvested at 10–14 dpp and 35–40 dpp, respectively. The cucumbers were peeled with a vegetable peeler, then quickly frozen in liquid nitrogen and stored in a refrigerator at −80 °C. A total of 4 groups of samples (YL14, OL14, YL19 and OL19), each group of samples prepared three biological repeats.

### 4.2. Determination of Total Flavonoids and Carotenoids

The average contents of carotenoids in dried peel of cucumber OL14 and OL19 were determined by Beijing Reagan Biotechnology Co., Ltd. Carotenoid detection kit (colorimetric method). According to Lambert–Beer’s law, the content of carotenoids can be calculated by crude extraction of carotenoids with organic solvents, that is, A = α CL (A: absorbance of the colored solution, C: concentration of the solution, L: thickness of the liquid layer, α: absorption coefficient). The total contents of flavonoids in cucumber OL14 and OL19 peel were determined by using the plant flavonoid test box of Nanjing Jiancheng Bioengineering Research Institute. In alkaline nitrite solution, flavonoids and aluminum ions form a red complex with a characteristic absorption peak at 502 nm. The content of flavonoids in the sample can be calculated by measuring the absorption value of the sample extract at 502 nm. Three biological repeats were carried out in each sample of the above experiment.

### 4.3. Sample Extraction and LC–MS/MS Analysis

The sample preparation, extract analysis, metabolite identification and quantification were performed at Wuhan MetWare Biotechnology Co., Ltd., Wuhan, China (www.metware.cn) following their standard procedures.

The freeze-dried peel was crushed using a mixer mill (MM 400, Retsch, Haan, German) with a zirconia bead for 1.5 min at 30 Hz. 100 mg powder was weighted and extracted overnight at 4 °C with 1.0 mL 70% aqueous methanol. Following centrifugation at 10,000 g for 10 min, the extracts were absorbed (CNWBOND Carbon-GCB SPE Cartridge, 250 mg, 3 mL; ANPEL, Shanghai, China, www.anpel.com.cn/cnw) and filtrated (SCAA-104, 0.22 μm pore size; ANPEL, Shanghai, China, http://www.anpel.com.cn/) before LC–MS analysis. The sample extracts were analyzed using an LC-ESI-MS/MS system (HPLC, Shim-pack UFLC SHIMADZU CBM30A system, Kyoto, Japan, www.shimadzu.com.cn/; MS, Applied Biosystems 6500 Q TRAP, Framingham, MA, USA, www.appliedbiosystems.com.cn/). The analytical conditions were as follows, HPLC: column, Waters ACQUITY UPLC HSS T3 C18 (1.8 µm, 2.1 mm×100 mm); solvent system, water (0.04% acetic acid): acetonitrile (0.04% acetic acid); gradient program, 100: 0 *v*/*v* at 0 min, 5:95 *v*/*v* at 11.0 min, 5:95 *v*/*v* at 12.0 min, 95:5 *v*/*v* at 12.1 min, 95:5 *v*/*v* at 15.0 min; flow rate, 0.40 mL/min; temperature, 40 °C; injection volume: 2 μL. The effluent was alternatively connected to an ESI-triple quadrupole-linear ion trap (Q TRAP)-MS.

LIT and triple quadrupole (QQQ) scans were acquired on a triple quadrupole-linear ion trap mass spectrometer (Q TRAP), API 6500 Q TRAP LC/MS/MS System, equipped with an ESI Turbo Ion-Spray interface, operating in a positive ion mode and controlled by Analyst 1.6.3 software (AB Sciex). The ESI source operation parameters were as follows: ion source, turbo spray; source temperature 500 °C; ion spray voltage (IS) 5500 V; ion source gas I (GSI), gas II (GSII) and curtain gas (CUR) were set at 55, 60 and 25.0 psi, respectively; the collision gas (CAD) was high. Instrument tuning and mass calibration were performed with 10 and 100 μmol/L polypropylene glycol solutions in QQQ and LIT modes, respectively. QQQ scans were acquired as MRM experiments with collision gas (nitrogen) set to 5 psi.DP and CE for individual MRM transitions was done with further DP and CE optimization. A specific set of MRM transitions were monitored for each period according to the metabolites eluted within this period.

### 4.4. RNA Extraction, cDNA Library Preparation and RNA Sequencing

Refer to the method of Miao et al. [1] to separating total RNA samples, after quality inspection (detection of pollution, degradation, etc.) is qualified, the residual DNA, was digested by DNase I and then rRNA was removed by Ribo-Zero kit. Fragmentation buffer was added to break RNA into 200–500 bp fragments in Thermomixer, and a strand of cDNA was synthesized with 6-base random primers (random hexamers) with short RNA as template. Using the first chain as the template, when synthesizing the second chain of cDNA, the cDNA synthesized by using dUTP instead of dTTP, was repaired by terminal repair and A was sequenced, and then UNG (Uracil-N-Glycosylase) was added to degrade the second chain. The fragment size was selected by agarose gel electrophoresis and then amplified by PCR. Finally, the library was sequenced by Illumina HiSeq X ten (Chengdu life baseline technology co., LTD, Chengdu, China). The whole set of annotated genes can be found in the National Center for Biotechnology Information (NCBI) SRA database (BioProject accession: PRJNA678478).

### 4.5. Analysis and Functional Annotation of Differentially Expressed Genes

After filtering and quality control of the original data obtained by sequencing, high-quality clean reads were obtained. The clean reads were compared to the reference genome sequence using HISAT2 software, and the comparison rate of each sample was calculated. We use the newly published Kallisto software [83] of NBT to determine which transcript reads comes from and quantify it accurately, filter the genes with low expression according to TPM < 1 and get the quantitative results of all sample gene levels. Based on the quantitative results of all genes in each sample, we used Noiseq software to screen differentially expressed genes between samples and filtered significant differentially expressed genes according to the threshold of |log2FC| ≥ 1 and probability >0.8. Finally, we analyzed the enrichment of GO and KEGG pathways in all DEGs and drew the corresponding network regulation pathway diagram (Figure 4).

### 4.6. qRT-PCR Verification of Gene Expression

Ten genes were selected by using qRT-PCR to verify the RNA-Seq results. All gene-specific primers were designed by Primer 5.0. The same RNA samples used in RNA-Seq were used in RT-qPCR. Each RNA sample was biologically repeated three times. The qRT-PCR reaction was performed by the DyNAmo Flash SYBR Green qPCR kit (Sagene Biotech, Guangzhou, China) and CFX96 qPCR system (Bio-Rad Laboratories, Shanghai, China). 10 µl reactions contained 2 µL of cDNA, 0.75 µl of each pair of target primers (200 nM), 1.5 µL of ddH2O and 5 µL of 2 × SYBR^®^ Green Supermix (Bio-Rad Laboratories, Shanghai, China). PCR conditions were as follows: 95 °C for 5 min (Preincubation); 40 cycles of 94 °C for 15 s and 60 °C for 45 s (2 step Amplification); 95 °C for 10 s, 65 °C for 60 s and 95 °C for 1 s (Melting); 37 °C for 30 s (Cooling). Relative gene expression levels were analyzed according to the 2∆∆Ct method. The internal normalization gene was *CsActin*. The gene-specific primers are listed in Supplementary Table S6. The details about the data collection are listed in Supplementary Table S7.

### 4.7. Statistical Analysis

The data of gene expression and metabolite were standardized to *Z* score by Log2. These data are used for the calculation of PCA and PCCs. We analyzed and compared the differences among four sample groups (YL14_vs_OL14, OL14_vs_OL19, YL19_vs_OL19, YL14_vs_YL19). The physiological data are expressed as the positive and negative standard deviations of the average values of the three repeated samples. SAS software was used for the analysis of variance. The difference between the two treatments was determined by the least significant difference test (*p* < 0.05). The numbers shown are generated by Microsoft Excel 2018.

## 5. Conclusions

For many years, because the yellow peel of cucumber and its corresponding mutants are difficult to obtain, or the collected materials are greatly affected by the environment, and their stability cannot be guaranteed, the research progress of peel yellow gene is slow. In this study, we analyzed the peel yellowing material L14 and its near-isogenic line L19 from the morphological, physiological, metabolic and transcriptional levels, and identified the genes and regulatory pathways related to cucumber peel yellowing. The results showed that the total amount of flavonoids in L14 old peel was significantly higher than that in L19 old peel. The results of association analysis between genes and metabolites and qRT-PCR verification showed that, *PAL* (*Csa6G147460.1*), *4CL* (*Csa5G154230.1*), *HCT* (*Csa7G431440.1*), *CCoAOMT* (*Csa7G073660.1*), *CHS* (*Csa3G600020.1*) and *FLS* (*Csa4G112650.1*) were candidate genes. Transcription factor coding genes *R2R3-MYB* (*Csa3G816030.1* and *Csa1G046820.1*), *bHLH51* (*Csa6G011720.1*) and *WRKY23* (*Csa6G139770.1*) may play an important role in affecting the yellowing of cucumber peel. On this basis, we also judged that the synthetic pathway of homoeriodictyol may play an important role in affecting the yellowing of cucumber peel. However, other key genes and fine regulatory networks of cucumber peel yellowing still need to be further explored and verified.

## Figures and Tables

**Table 1:** The differentially accumulated flavonoids (DAFs) related to peel yellowing in L14 and L19.

Component Name	Compounds	OL14 vs. OL19 LogFC	YL14 vs. OL14 LogFC	Pathway ID	Index
Isoflavone					
	6-Hydroxydaidzein	−14.12976	14.12976	c	pme3261
	Glycitein	−11.50735	11.50735	c	pme3251
	Calycosin	−11.45121	11.45121	c	pme3230
	Formononetin 7-O-glucoside (Ononin)	−10.78560	10.78560	c	pme3502
	Genistein 7-O-Glucoside (Genistin)	−8.44094	9.46536	c	pme3210
Flavanone					
	Naringenin O-malonylhexoside	−17.18100	17.18100	-	pma0791
	Naringenin	−8.77594	10.47588	a, c	pme0376
	Naringenin chalcone	−6.71026	6.83489	a	pme2957
	Homoeriodictyol	−6.07142	6.00144	a	pme3461
	Hesperetin	−5.73054	6.56258	a	pme2319
	Isosakuranetin (4’-Methylnaringenin)	−3.78556	8.45274	-	pme3464
Flavone C-glycosides					
	Apigenin C-glucoside	−16.90395	5.52477	-	pma1108
	Isovitexin	−13.37429	4.78364	d	pme0374
	Naringenin C-hexoside	−12.92101	3.95331	-	pma0724
	C-hexosyl-apigenin O-caffeoylhexoside	−11.33398	2.92648	-	pma6254
	C-hexosyl-apigenin O-p-coumaroylhexoside	−7.01972	4.84490	-	pmb0680
	Apigenin 6-C-hexosyl-8-C-hexosyl-O-hexoside	−5.59166	3.92600	-	pmb0613
	8-C-hexosyl-apigenin O-hexosyl-O-hexoside	−5.43549	3.87321	-	pmb0639
Flavonol					
	Kaempferide	−15.43731	15.43731	d	pma1116
	Aromadedrin (Dihydrokaempferol)	−13.13512	13.13512	a	pme2963
Flavone					
	Acacetin O-acetyl hexoside	−12.48981	12.48981	-	pmb2987
	sakuranetin	−11.85475	11.85475	-	pme1662
	Butin	−8.86582	10.58564	a	pme3473
	Apigenin 5-O-glucoside	−8.47617	19.03466	-	pme0359
	Apigenin 7-O-glucoside (Cosmosiin)	−8.05708	8.32675	d	pmb0605
	Apigenin	−4.35888	4.98833	a, c, d	pme0379
Anthocyanins					
	Pelargonidin 3-O-beta-D-glucoside (Callistephin chloride)	−14.15048	5.65299	-	pme3392
	Pelargonidin	−3.76095	14.92857	a, b	pme1397
Catechin derivatives					
	Epicatechin gallate (ECG)	−10.38881	10.38881	-	pme1562
	Epigallate catechin gallate (EGCG)	−9.86568	1.90675	-	pme1486

Pathway ID: a = ko00941; b = ko00942; c = ko00943; d = ko00944.

**Table 2:** Differential expression genes (DEGs) related to peel yellowing in OL14_vs_OL19.

GeneID	OL14_TPM	OL19_TPM	Log_2_FC	Description
*Csa6G147460.1*	14.38	2.82	−2.35	Phenylalanine ammonia-lyase, PAL
*Csa6G446290.1*	29.73	14.41	−1.05	Phenylalanine ammonia-lyase, PAL
*Csa3G710200.1*	27.32	3.75	−2.86	4-coumarate-CoA ligase, 4CL
*Csa5G154230.1*	149.94	65.11	−1.20	4-coumarate-CoA ligase, 4CL
*Csa7G431440.1*	11.08	1.86	−2.57	Shikimate O-hydroxycinnamoyltransferase, HCT
*Csa7G073660.1*	131.04	18.06	−2.86	Caffeoyl-CoA O-methyltransferase, CCoAOMT
*Csa3G600020.1*	78.22	10.12	−2.95	Chalcone synthase, CHS
*Csa4G112650.1*	15.13	4.97	−1.60	Flavonol synthase, FLS

## Data Availability

Data available in a publicly accessible repository that does not issue DOIs Publicly available datasets were analyzed in this study. This data can be found here: [https://www.ncbi.nlm.nih.gov/bioproject/PRJNA678478 / BioProject accession: PRJNA678478].

## Supplementary Materials

The following are available online at https://www.mdpi.com/1422-0067/22/3/1494/s1, Figure S1: Wego map of differentially expressed genes, Table S1: Summary table of flavonoid metabolites, Table S2: Statistical table of DEGs in four groups of L14 and L19, Table S3: Enrichment results of differential gene pathway, Table S4: Statistical table of TFs in four groups of L14 and L19, Table S5: TFs classification table of L14 and L19, Table S6: The gene-specific primers. Table S7: The qPCR data collection results.
