# Supplementary figures and images for: Integrated Metabolome and Transcriptome Analysis Unveils Novel Pathway Involved in the Formation of Yellow Peel in Cucumber
Source: Int J Mol Sci. 2021 Feb 2;22(3):1494. doi: 10.3390/ijms22031494 (PMC7867363; doi:10.3390/ijms22031494)

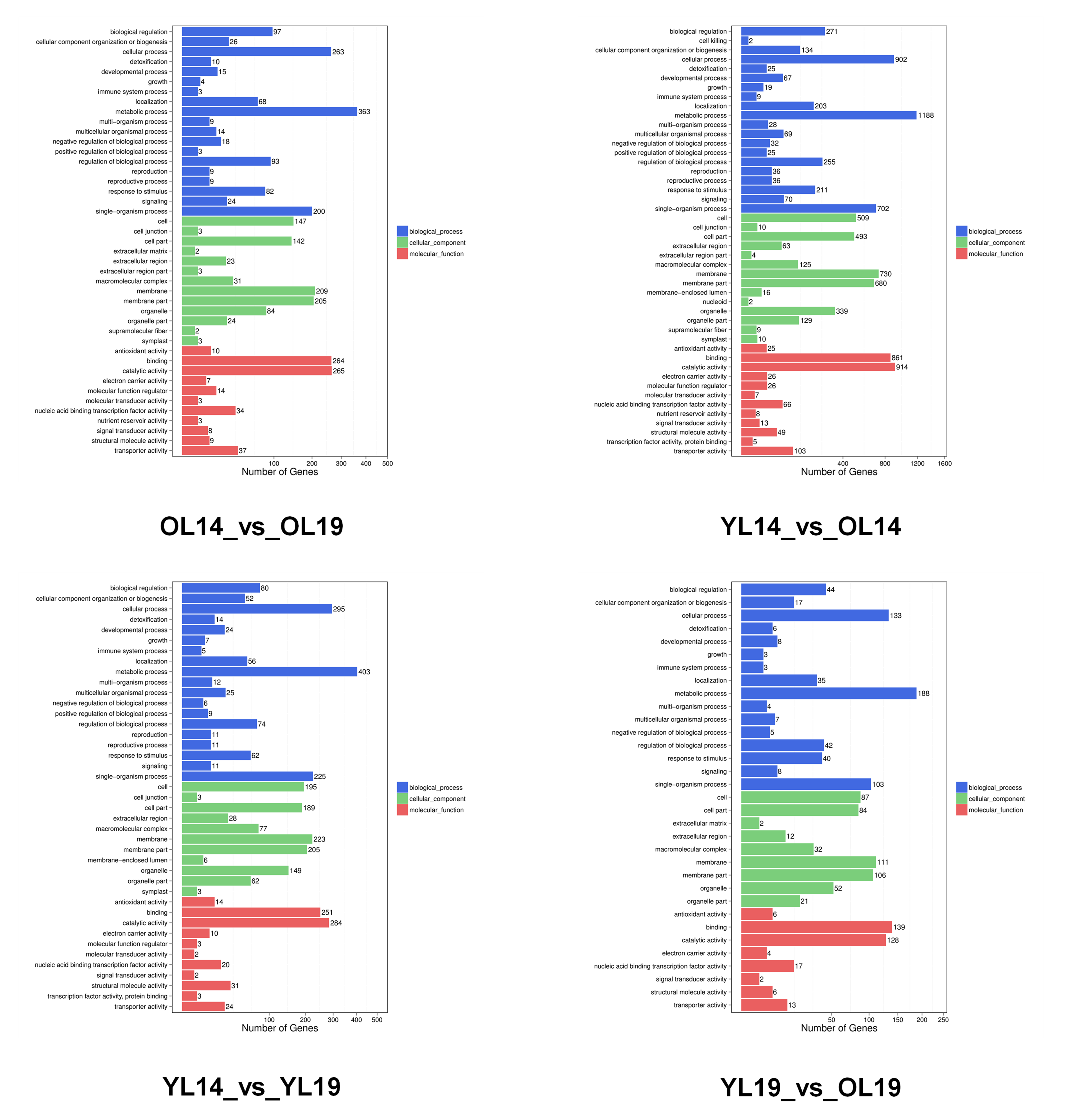

Supplement: Supplementary file 1 [file ijms-22-01494-s001.zip › ijms-1074512-proof done sup/Figure S1. Wego map of differentially expressed genes.tif]
